# Validity and reliability of automated treadmill six-minute walk test in patients entering exercise-based cardiac rehabilitation

**DOI:** 10.1080/07853890.2024.2304664

**Published:** 2024-01-17

**Authors:** Marketa Nevelikova, Filip Dosbaba, Garyfallia Pepera, Marian Felsoci, Katerina Batalikova, Jing Jing Su, Ladislav Batalik

**Affiliations:** a Department of Rehabilitation, University Hospital Brno, Brno, Czech Republic; b Department of Rehabilitation and Sports Medicine, 2nd Faculty of Medicine, Charles University, University Hospital Motol, Prague, Czech Republic; c Department of Physiotherapy and Rehabilitation, Faculty of Medicine, Masaryk University, Brno, Czech Republic; d Clinical Exercise Physiology and Rehabilitation Research Laboratory, Physiotherapy Department, School of Health Sciences, University of Thessaly, Lamia, Greece; e Department of Internal Medicine and Cardiology, University Hospital Brno, Brno, Czech Republic; f School of Nursing, The Hong Kong Polytechnic University, Hong Kong, China

**Keywords:** Six minute walk test, cardiac rehabilitation, automatized treadmill, functional capacity, technology-assisted assessment

## Abstract

**Introduction:** The six-minute walk test (6MWT) is a well-established tool for assessing submaximal functional capacity for cardiac patients, but space limitations challenge its implementation. Treadmill-based (TR) 6MWT is a promising alternative, but it requires patients to complete a familiarization test to adapt treadmill speed regulation. With the advancement of sensors, it is possible to automatically control speed for individual patients and thus overcome the space limitation or the speed control difficulty on the treadmill for each patient.

**Methods:** This study investigated the validity and interchangeability of automated speed TR6MWT and standard hallway (HL) 6MWT. Eighteen patients were assessed at baseline of the 12-week cardiac rehabilitation program. Fourteen of them were assessed after rehabilitation. All patients performed three TR6MWTs and three HL6MWTs at baseline and one of each test after the program.

**Results:** Patients well tolerated the TR6MWT. There was a strong correlation between both test methods (r = 0.79). However, patients performed significantly better in HL6MWT (514.8m ± 59.7m) than in TR6MWT (447.2 ± 79.1m) with 95% CI, 40.4-94.6m, p < 0.05. Both tests showed high test-retest reliability (intraclass correlation coefficient of 0.86). The TR6MWT showed a valuable comparison of the effect of the cardiac rehabilitation program (20% increase, effect size 1.1) even though it is not interchangeable with the HL6MWT.

**Conclusion:** The automated speed TR6MWT appears to be an acceptable tool with adequate validity, reliability, and responsiveness for assessing functional capacity in patients utilizing cardiac rehabilitation programs.

## Introduction

Walking tests are practical tests of functional capacity in patients with cardiorespiratory diseases [1–3]. The six-minute walk test is the most widely used (6MWT) [4,5]. This test assesses the submaximal level of functional capacity by measuring the distance a patient can walk on a flat surface quickly for six minutes. The 6MWT is considered a safe alternative to the cardiopulmonary exercise test for cardiopulmonary risk stratification and to assess functional capacity for measuring the effect of exercise-based interventions [6].

The 6MWT is a valid and reliable tool for assessing the functional capacity of patients with cardiorespiratory disease in clinical practice and research studies. The 6MWT score correlates with maximal power output achieved during the cycle-ergometer cardiopulmonary exercise test during cardiac rehabilitation and is strongly correlated with peak oxygen consumption (*r* = 0.82, *p* < 0.001) in patients with cardiovascular or respiratory disease [7,8]. Reproducibility of the 6MWT is generally high (with an intraclass correlation coefficient above 0.9) [9,10]. Reproducibility of the result for the ability to detect a change and suggested considering a 44 m distance difference as the minimum allowing to interpret as significant a variation between two consecutive tests performed the same day. In patients participating in exercise-based cardiac rehabilitation, a 2–8% learning effect was shown between repeated tests [11]. In addition, there was substantial evidence for the potential of 6MWT to demonstrate a change in clinical status. Bellet et al. found a mean difference in 6MWD distance after a cardiac rehabilitation program of 60.4 m (95% confidence interval (CI) 54.5 − 66.3 m) with a mean effect size of 0.96 [11].

However, space limitations challenge the implementation of 6MWT. The guidelines for conducting this hallway test (HL6MWT) recommend a straight indoor corridor with a hard surface of 30 meters [12], which is not always available, and many clinical rehabilitation centers have limited space for testing. The treadmill testing alternative to the 6MWT (TR6MWT) provides space savings and allows for stable monitoring of exercise capacity [13,14]. The TR6MWT has been demonstrated as a substitution tool with adequate validity that is well tolerated and sensitive to assessing functional capacity in patients after cardiac surgery [14]. A limitation of the TR6MWT is the need to complete a familiarization test to adapt treadmill speed regulation.

The average reported difference in the comparison between the TR6MWT and HL6MWT is high (range: 50–102 m); thus, tests cannot be used interchangeably. For this reason, treadmill use was not recommended in the approved guidelines for 6MWT [15].

Technological developments in sensors have created innovative possibilities for automated speed control on the treadmill. Therefore, automated speed control could be more responsive to individual patient needs and overcome the practical limitations of the HL6MWT. To the best of our knowledge, the validity and reliability of automated TR6MWT have not yet been studied in patients entering cardiac rehabilitation. This study aimed to investigate the validity and interchangeability of automated TR6MWT and standard HL6MWT. A secondary aim was to determine the applicability of automated TR6MWT to measure the effect of the cardiac rehabilitation program.

## Methods

### Participants

Patients were recruited from those entering a standard cardiac rehabilitation program (phase II) at the cardiology clinic of the University Hospital Brno between 1 June 2022 and 1 April 2023. The study complied with the Declaration of Helsinki, and the protocol was approved by the Ethical Committee of the University Hospital Brno (protocol number 07-090621/EK, approval date on 9 June 2021). The inclusion criteria consisted of diagnosis of acute myocardial infarction, percutaneous coronary intervention, or aortocoronary bypass graft in the last three months, indication for an exercise-based cardiac rehabilitation program by a clinical cardiologist, treatment with the recommended pharmacological therapy (including beta blockers therapy [16]), ability to walk on a treadmill without risk of falling, age above 18, signed informed consent with participation in the study. Exclusion criteria included significant cardiovascular risk, orthopedic pathologies, and neurological disorders affecting gait.

### Assessment

**Figure 1.**
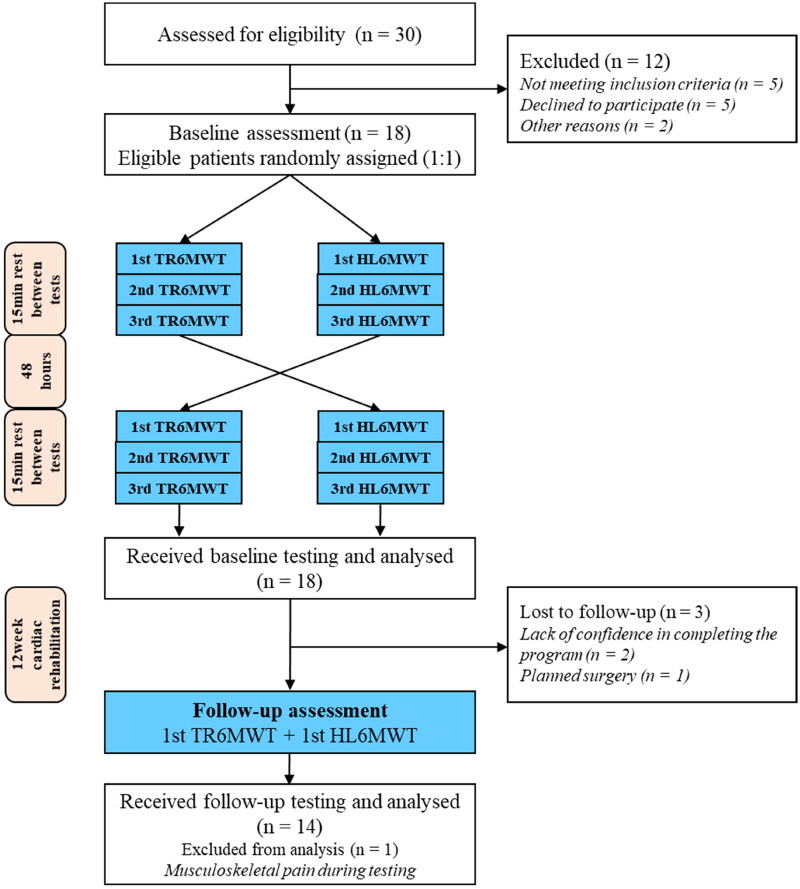
Flowchart of the study.

Patients were tested with two different walking tests at the beginning and the end of the 12-week exercise-based program. All patients performed three TR6MWTs and three HL6MWTs at baseline and one of each test after the program (Figure 1).

**Figure 2.**
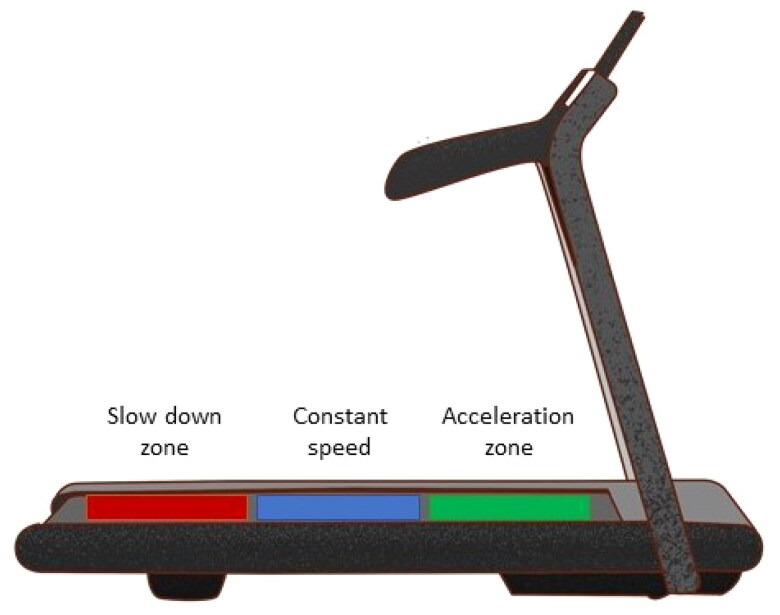
Treadmill with automatized gait speed function.

The standard HL6MWT represented a marked-out 30 m course on a straight corridor. Before starting, patients were instructed to warm up by walking two times 30 m. A standard HL6MWT was then performed following the guidelines [15]. Patients were given instructions detailed in supplementary file 1. The experimental automated TR6MWT was performed on a commercially available treadmill trainer (EN-MOTION Enraf-Nonius, Netherlands). The total test distance walked was displayed on the device monitor. The treadmill has built-in sensors with high distance resolution (Figure 2), allowing speed automation. The automated speed function means that the speed of the treadmill automatically adapts to the pace of the person using the treadmill. The walk speed is automatically reduced if that person cannot maintain the previously selected speed. If the person can walk faster, the speed will automatically increase. The speed change was adaptable in a 0.1–0.5 km/h/s range.

Patients were instructed the same way about the test task as in the standard HL6MWT protocol [15]. Patients were instructed on how to stop if needed. Patients were allowed to hold on to the handrail of the treadmill for safety. The rest of the testing was the same, including a warm-up walk in an auto-speed version that was comfortable while walking on the treadmill. A short break was due after the warm-up phase. The test was not started if patients reported discomfort or movement limitations during the 2-min warm-up phase. Two physiotherapists experienced in clinical testing for exercise-based programs were present during testing, and a cardiologist was available in the clinical ward for emergencies. The distance covered was not reported to the patients.

At the end of each test was measured the total distance walked, the rating of perceived exertion (Borg scale: 7–20) and peak heart rate using a wearable sensor and heart rate monitor (Polar H10, M430 Kempele, Finland)

### Cardiac rehabilitation programme

The exercise-based CR program was set for 12 week period. Patients were prescribed three exercises per week according to guidelines [17]. Exercise prescription consisted of individualized graded aerobic exercise on aerobic equipment (ergometer, treadmill, rowing machine) at an intensity of 60–80% of predicted maximum heart rate (HRmax) according to the recommended formula: HRmax = 164 − 0.7 × age. Duration of exercise was individually prescribed on 30–60 min per session [18]. The program was led by a physiotherapist at a rehabilitation clinic.

### Statistics

In this study, we conducted various analyses to examine different aspects of the longest walking distances. To assess the data distribution, we employed the Shapiro-Wilk test. The agreement between the walking tests was analyzed using the Bland-Altman method. Interpretation of the correlation coeficient represents values of less than 0.5 indicating poor reliability; values between 0.5 and 0.75, indicating moderate reliability; values between 0.75 and 0.9, indicating good reliability; and values greater than 0.9, indicating excellent reliability. Furthermore, we investigated the correlation between the walk tests using Pearson’s correlation coefficient. To compare the longest walk distances obtained on each test method, as well as the peak heart rate differences between pre-test and post-test heart rates, on each test day, we utilized the paired T-test. The Borg rating of perceived exertion was compared using the Wilcoxon test for paired samples.

In the assessment of reliability, the test-retest reliability of both the TR6MWT and HL6MWT was examined at baseline of the cardiac rehabilitation program. This evaluation encompassed four key aspects: the presence of systematic error, relative reliability, absolute reliability, and minimal detectable change. The identification of systematic error among the three 6MWTs was accomplished through the utilization of an analysis of variance for repeated measurements. Intraclass correlation coefficients exceeding 0.75 were deemed indicative of good reliability [19]. Absolute reliability was ascertained by employing the standard error of measurement (SEM), which was estimated as the square root of the mean-square-error term derived from the analysis of variance. The SEM served as a representation of the standard deviation of the patients’ scores during repeated testing. Furthermore, the minimal detectable change was calculated based on the SEM using the formula: MDC = 1.96 × SEM × square root of 2. In terms of statistical analysis, the concurrent validity of the TR6MWT was evaluated by considering the 6MWT as the gold standard, and subsequently determining the Pearson correlation (r) between the best performances on the two tests. This correlation was computed at baseline of the cardiac rehabilitation.

To evaluate the effectiveness of the exercise-based cardiac rehabilitation intervention, two approaches were employed to assess the TR6MWT and the HL6MWT. Firstly, a paired t-test was conducted to compare the best performances achieved at baseline and after the 12-week cardiac rehabilitation program for both tests. This statistical test allowed for the examination of any significant differences between the pre-and post-intervention measurements. Secondly, the effect size was calculated as a measure of the magnitude of change observed in the mean scores relative to the standard deviation of the baseline scores. The effect size indicates the practical significance of the intervention. Effect sizes of 0.8 or higher are considered significant, effect sizes ranging from 0.5 to 0.8 are considered moderate, and effect sizes between 0.2 and 0.5 are considered minor [20]. All statistical calculations were performed using statistical software Statistica 12 (TIBCO, Software Inc, Palo Alto, CA, USA).

The sample size was calculated by considering the identification of a moderate correlation (0.65) between the distances covered in the TR6MWT and the HL6MWT, with a power of 85% and a significance level of 0.05 [14]. The study determined that 18 patients would be required.

## Results

**Figure 3.**
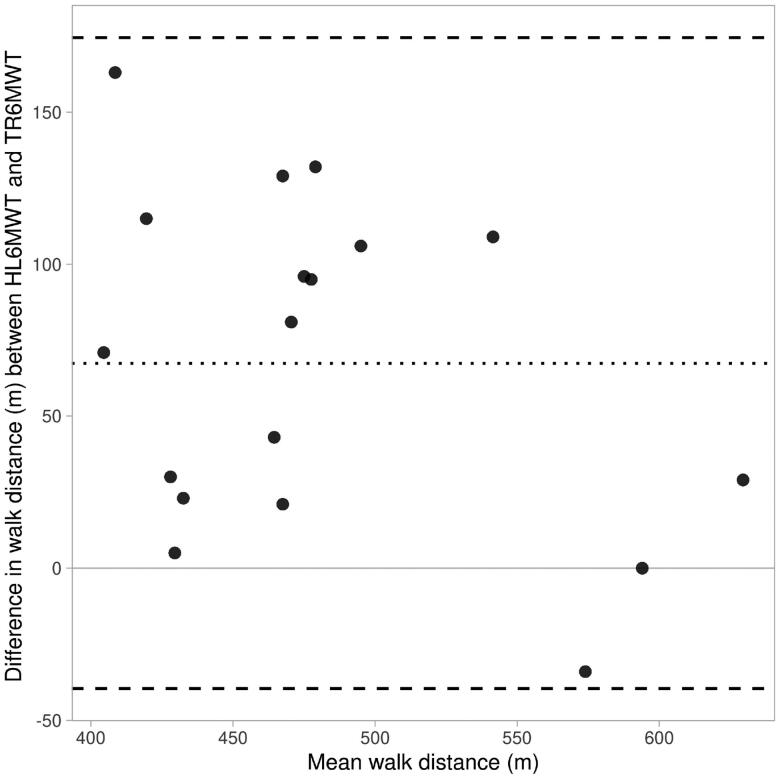
Limits of agreement plot.

Table 1 represents the 18 patient’s characteristics, and Figure 1 the study flow. The mean HL6MWT result (514.8 *m* ± 59.7 m) was significantly higher than 447.2 ± 79.1 m measured from TR6MWT. Patients achieved a mean performance of 79.3 ± 11.8% of the reference value [21]. Fifteen patients (83%) of the 18 patients completed a greater distance on the HL6MWT than TR6MWT. The Bland-Altman plot in Figure 3 indicates a lack of agreement between the tests. The difference in distance between walking in the hallway and on the treadmill tended to decrease as the average distance increased, as demonstrated by the regression line. Of the 18 patients, nine (50%) had differences in hallway versus treadmill walk distance greater than 10% 67 m.

**Table 1.** Characteristics of study patients (*n* = 18).

Age (yr)	52.1 ± 10.3
Male/Female	13/5
Weight (kg)	88.6 ± 13.5
Height (cm)	173.3 ± 8.7
Body mass index (kg/m2)	29.3 ± 4.0
Ejection fraction (%)	54.4 ± 7.0
Diagnosis acute myocardial infraction (n)	18
Duration after diagnosis (weeks)	5.9 ± 2.5
Percutaneous coronary intervention (n)	18

Values are expressed as means ± standard deviation.

Graph that shows the data as dots (visibility: 0.8). The dotted line indicates the average difference of 67.4 m, and the dashed lines indicate the Limits of Agreement: −39.6 m & 174.5 m. The Shapiro-Wilk test of the difference returns a p-value of 0.68, which does support a normal distribution.

The analysis of variance for repeated measures demonstrated a statistically significant difference for each walking test administered before cardiac rehabilitation (*p* < 0.001). The reliability indexes for the HL6MWT were calculated as an intraclass correlation coefficient (ICC) of 0.86 (95% confidence interval CI 0.82–0.89), while TR6MWT, the ICC was 0.84 (95% CI 0.78–0.87). Notably, the standard error of measurement (SEM) was found to be higher for the TR6MWT compared to the HL6MWT with values of 41 m and 60 m, respectively. Furthermore, the SEMs represented 8% of the mean distance covered during the HL6MWT and 13% of the mean distance covered during the TR6MWT. Additionally, the minimal detectable change (MDC) for the HL6MWT was determined to be 113 m, while for the TR6MWT, it was 166 m.The correlation between the maximum walking distances in the HL6MWT and TR6MWT was found to be strong (*r* = 0.79), and this difference was statistically significant (*p* < 0.05). Consequently, the probability of accurately predicting the distance walked in the hallway based on the distance walked on the treadmill is only 63%, which is considered unsatisfactory. Additionally, a notable learning effect was observed in the second and third tests in TR6MWT (5.4 and 3.6%) and in HL6MWT (4.9 and 3.1%). In both, there were significant differences between all three tests. In more than half of the cases, the best performance for both methods was in the third test. In the HL6MWT, the most significant walk distance was in the third test in 12 patients and the TL6MWT in 10 patients. The first test was the best performance only in two cases in both tests.

**Figure 4.**
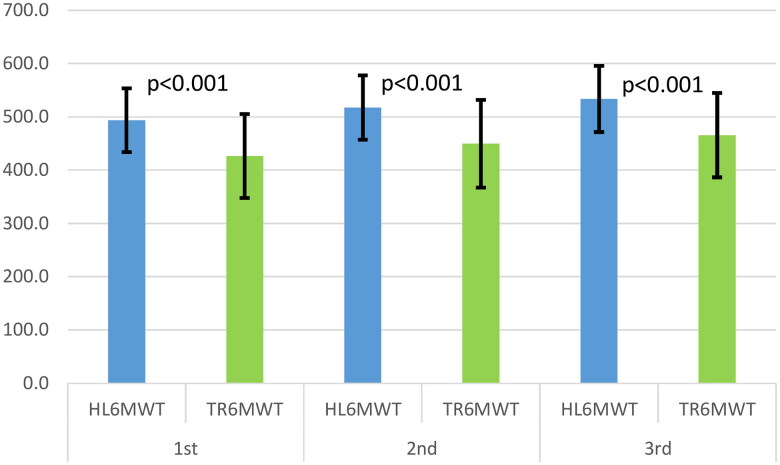
Mean walking distance covered during each round of testing.

The walking distance change between the HL6MWT and TR6MWT did not tend to decrease from the first to the third test (Figure 4). There were no significant differences in the peak heart rate after each test (*p* = 0.59). Most patients (12/18) reached their peak heart rate after the HL6MWT. There was also no statistically significant difference in the rating of perceived exertion (*p* = 0.50). Eight subjects reported more significant dyspnea after the treadmill test.

No serious adverse events were reported during testing. In one case, after the cardiac rehabilitation program, the patient reported musculoskeletal pain during TR6MWT, limiting achieving testing performance.

## Results after cardiac rehabilitation program

Fifteen patients (83%) completed the exercise-based cardiac rehabilitation program. Patients attended 28.4 ± 5.5 exercise sessions (78.9% of the total, 12–35 sessions). Fourteen patients completed the final testing. The mean HL6MWT distance was significantly higher (589.4 ± 55.0 m by 14.5% *p* < 0.01) compared to baseline testing. The mean TR6MWT distance was significantly higher (536.4 ± 77.9 m by 19.9% *p* < 0.01) compared to baseline testing. Effect sizes were 1.3 for HL6MWT and 1.1 for TR6MWT.

## Discussion

A modified version of the standard HL6MWT, the TR6MWT, was developed as an alternative protocol. In our study, we aimed to evaluate the feasibility of the TR6MWT and compare its results to those obtained from the standard HL6MWT. The TR6MWT protocol was practical to administer and was well-acceptable by all study participants. Nevertheless, we observed a significant change between the two tests, with the larger number of patients walking a superior distance in the HL6MWT. This aligns with previous research findings, suggesting that the TR6MWT and HL6MWT are not interchangeable and may yield different results [22,23]. However our study confirmed the validity and reliability of using treadmill to administer the 6MWT by presenting good reliability values in the TR6MWT and HL6MWT assessments (ICC: 0.75–0.9). Previous studies shows the treadmill as valid and reliable tool compared to HL6MWT assessment. Especially, in patients after cardiac surgery and acute coronary syndrome, there presented good [14] and excellent [23] reliability results for the TR6MWT (ICC > 0.90) [20].

Several cases describe the discrepancies in performance between the two tests. The two tests may need various skills required. Some patients are more comfortable with treadmill usage and automatized gait speed than others. Walking in a hallway is a daily skill and, therefore, more practical to patients than walking on a treadmill. On the other hand, adaptation to a new motor function differs for each patient and takes a different amount of time. Patient motivation is also debatable, as it may differ between the two tests. Even though the instructions were similar for both tests, the encouragement may likely be higher with the HL6MWT, which is more natural and motorically familiar to the patient. Previous research has shown encouragement during the 6MWT positively influenced results by 30 m [24].

Another case is the slow response of the automated treadmill function to adjust the baseline gait acceleration, which started at zero. It seems more advantageous to use the initial velocity. As stated by Pieter-Henk Boer, the protocol started at an initial velocity of 4 km/h [25]. Further studies may recruit more participants to establish cut-off walking distance for the TR6MWT, considering that patients tend to perform better when walking in a hallway than on a treadmill.

Next, the hypothesized correlations between the maximum distance walked during the HL6MWT and TR6MWT were noted regarding validity. These results support the acceptable concurrent validity of the TR6MWT, similar to the research by Olper et al. [14] and Jensen et al. [23], who demonstrated significant correlations between the two tests in cardiac patients.

Laskin et al. found that between three sets of TR6MWT and HL6MWT, there were no statistically significant differences in perceived exertion or peak heart rate ratings [26]. This is consistent with our study, where we did not find significant differences in peak heart rate or ratings of perceived exertion, which we hypothesized were measures of effort for patients. The result suggests that patients made similar efforts during the hallway and treadmill tests. For the experimental automated TR6MWT, this is a potential practical utility in medical treatment for delivering measurable results and indicating a functional response.

Learning effects are well-known in HL6MWT [27]. In our study, a learning effect was observed for both the hallway and treadmill tests and was more significant for the TR6MWT. Previous studies have shown a significant increase in HL6MWT distance measured over several tests [22,27]. Most of the difference occurred during the first three tests [22]. We assessed the reproducibility of TR6MWT and compared the results with HL6MWT reproducibility. The overall variability of the three distances was similar, and a nonsignificant increase was found between the first and second hallway and treadmill or between the second and third hallway and treadmill 6MWT.

In the walk test, the three tests minimized the effect of improvement in walking distance [28]. Our results suggest that the use in clinical practice of at least two TR6MWT should be performed to eliminate a learning effect and ensure measurement accuracy [29].

The result of the current research regarding the increase in the average distance walked in the TR6MWT showed an improvement in the functional capacity of patients after an exercise-based cardiac rehabilitation program. Functional capacity is one of the crucial factors determining patient prognosis [26]. Our results of 19.9% (89.2 m) are consistent with a study by Peixoto et al. who reported a significant increase in functional capacity of 20% (85 m) in patients undergoing exercise training after acute myocardial infarction [30]. Despite the lack of agreement between the HL and TR6MWT, which limits the interchangeability of the tests, our study strength is the practical benefit of the TR6MWT because it showed a valid assessment of the cardiac rehabilitation therapy effect. The utilization and analysis of the results from the 6MWT can provide valuable guidance for clinical decision-making. It is crucial to ascertain whether a specific intervention has resulted in any meaningful alteration in the patient’s functional capacity. Substantial evidence supports the notion that alterations in performance on the 6MWT following a cardiac rehabilitation program indicate changes in the patient’s clinical functional status [31,32]. Moreover, Papathanasiou et al. showed that clinically significant increments in mean distance covered in a 6MWT following a cardiac rehabilitation program in patients with ischemic heart disease are around 70–170 m [33], which is consistent with the results of the present study.

Digital technologies represent significant potential for developing and facilitating cardiac rehabilitation [34–36]. The benefits derived from current research on technology-assisted testing represent a space-saving over standard HL6MWT or cardiopulmonary exercise testing examinations. Currently, healthcare providers and their limited economic budgets are seeking cost savings. Thus, future research needs to incorporate cost-effectiveness outcomes to allow assessment of cost savings of a technology-assisted TM6MWT and HL6MWT option.

The board of outpatient cardiac rehabilitation providers often does not have the space available for functional testing. Therefore, the technology-assisted TR6MWT may represent an effective and safe alternative. In addition, patients unable to walk separately for 6 min can perform this test using a treadmill with a reduced risk of falls. However, along with digital and technological developments, there is a need to clarify the indications and use of monitoring technologies provided by the devices.

## Limitations

One limitation of the current study was the use of a randomized process, which may have influenced the reliability of the TR6MWT results. This is because patients who performed the HL6MWT first may have experienced a training effect, leading to potentially improved performance in the subsequent TR6MWT conducted 48 h later. Another area for improvement is the use of single-site sampling, which implies a potential weakness in the generalizability of the study results to other contexts. To align the results with the clinical care situation, the tests were administered by two physiotherapists who specialized in working with cardiac patients. However, it is essential to note that this approach may have introduced increased variability between the tests [37]. Finally, turning in the traditional 6MWT may affect the outcome of the test in some less-able patients with reduced mobility and greater orthopedic limitations. Compared to the TR6MWT, this limitation is not present, which may have biased the study results.

## Conclusion

The feasibility and safety of conducting the automatized TR6MWT have been demonstrated in cardiac rehabilitation patients. TR6MWT showed a valuable comparison of the effect of the cardiac rehabilitation program. However, it is essential to note that the results obtained from the HL6MWT and TR6MWT cannot be used interchangeably. This is due to the significant personal variability observed, resulting in a high disagreement between the walking distances in the two test scenarios.

## Supplementary Material

Supplemental Material
