# Supplementary material for: Validity and reliability of automated treadmill six-minute walk test in patients entering exercise-based cardiac rehabilitation
Source: Ann Med. 2024 Jan 17;55(2):2304664. doi: 10.1080/07853890.2024.2304664 (PMC10798278; doi:10.1080/07853890.2024.2304664)
Supplement: Supplemental Material [file IANN_A_2304664_SM2121.docx]

Guidelines and Motivation for the Six-Minute Walk Test in the Hallway

Begin by informing the participant in the following manner:

"For this assessment, your objective is to cover as much distance as you can within 6 minutes. Your walking route will involve moving back and forth along this corridor. Given the duration, you will be engaging in a sustained effort, likely experiencing breathlessness or fatigue. Feel free to reduce your pace, come to a complete stop, and take breaks whenever necessary. While resting, leaning against the wall is allowed, but resume walking as soon as you can."

"As you navigate the course, you will encounter cones. When approaching them, execute brisk pivots and continue in the opposite direction without delay. Allow me to demonstrate one lap myself – walking and pivoting around a cone."

"Are you prepared to proceed with the test? I'll use this counter to record the number of laps you accomplish. Each time you reach this starting line, I will click the counter. Remember that the goal is to cover as much ground as possible in 6 minutes, but refrain from running or jogging."

"Whenever you feel ready, you can commence the test."

Encouragement Phrases:

After the first minute:

"You're doing great. You still have 5 minutes to go."

At 4 minutes remaining:

"Keep up the good work. You have 4 minutes left."

When there are 3 minutes left:

"You're doing well. You're halfway through; just 3 more minutes to go."

With 2 minutes remaining:

"Keep it up. Only 2 minutes left."

When there's only 1 minute left:

"Fantastic effort. Just 1 more minute to go."

In the event, the participant stops and needs to rest:

"Feel free to lean against the wall if needed. Resume walking whenever you're ready. I won't stop the timer."

If the participant decides to discontinue before 6 minutes:

"If you choose not to continue, we can provide a chair. Please note the distance, time stopped, and the reason for discontinuation."

15 seconds from completion:

"In a moment, I'll ask you to stop. When I do, pause right where you are, and I'll come over to you."

When the timer rings or buzzes:

"Stop!" Walk over to the participant, considering a chair if they seem exhausted. Mark the stopping point with a bean bag or tape.

Guidelines and Motivation for the Treadmill Six-Minute Walk Test

Inform the participant with the following instructions:

"The objective of this test is to walk as far as you can within a 6-minute duration on a treadmill. Throughout the test, support yourself using the treadmill handrails. You can monitor and adjust the speed yourself, but if you feel uneasy, I can assist you upon your request."

"At any point, feel free to modify the speed as needed. Given the extended duration, be prepared for exertion, and you may experience breathlessness or fatigue."

"You are allowed to decrease speed, come to a stop, or take breaks as required. While resting, you may lean against the treadmill handrails, but resume walking as soon as possible."

"Are you prepared to begin?"

"I will use the treadmill monitor to record the distance covered. Once the test concludes, I will provide you with the distance walked."

"Remember that the goal is to cover as much distance as possible within 6 minutes without running or jogging."

"Feel free to commence whenever you are ready."

Encouragement Phrases:

After the first minute:

"You're making good progress. You still have 5 minutes to go. Remember, you can adjust the treadmill speed at any time."

At 4 minutes remaining:

"Keep up the excellent effort. You've got 4 minutes left. Feel free to modify the treadmill speed whenever needed."

When there are 3 minutes left:

"You're doing great, and you're halfway through. Keep it up! Feel free to adjust the treadmill speed as required."

With 2 minutes remaining:

"Keep going strong. Just 2 minutes left. And don't forget, you can adjust the treadmill speed whenever needed."

When there's only 1 minute left:

"Fantastic job! Only 1 more minute to go. Remember, you can control the treadmill speed as needed."

If the participant stops and needs to rest:

"Feel free to lean against the treadmill handrails if you'd like. Let me know when you're ready to continue. Don't worry; I won't stop the timer."

If the participant stops before 6 minutes:

"If you choose not to continue, we can provide a chair. Please note the distance, time stopped, and the reason for discontinuation."
